# The test–retest reliability of physiological and perceptual responses during treadmill load carriage

**DOI:** 10.1007/s00421-024-05435-0

**Published:** 2024-02-28

**Authors:** Danielle M. Vickery-Howe, Ben J. Dascombe, Anthea C. Clarke, Jace R. Drain, Minh Huynh, Kane J. Middleton

**Affiliations:** 1 Sport, Performance, and Nutrition Research Group, School of Allied Health, Human Services and Sport, La Trobe University, Melbourne, Australia; 2 School of Health Sciences, Western Sydney University, Campbelltown, Australia; 3 Applied Sport Science and Exercise Testing Laboratory, School of Life and Environmental Sciences, University of Newcastle, Ourimbah, Australia; 4 Human and Decision Sciences Division, Defence Science and Technology Group, Fishermans Bend, Australia

**Keywords:** Repeatability, Walking, MetaMax, Heart rate, Military

## Abstract

**Purpose:**

Understanding the test–retest reliability of physiological responses to load carriage influences the interpretation of those results. The aim of this study was to determine the test–retest reliability of physiological measures during loaded treadmill walking at 5.5 km h^−1^ using the MetaMax 3B.

**Methods:**

Fifteen Australian Army soldiers (9 male, 6 female) repeated two 12-min bouts of treadmill walking at 5.5 km h^−1^ in both a 7.2 kg Control condition (MetaMax 3B, replica rifle) and a 23.2 kg Patrol condition (Control condition plus vest) across three sessions, separated by one week. Expired respiratory gases and heart rate were continuously collected, with the final 3 min of data analysed. Ratings of Perceived Exertion and Omnibus-Resistance Exercise Scale were taken following each trial. Reliability was quantified by coefficient of variation (CV), intra-class correlation coefficients (ICC), smallest worthwhile change (SWC), and standard error of the measurement.

**Results:**

Metabolic and cardiovascular variables were highly reliable (≤ 5% CV; excellent-moderate ICC), while the respiratory variables demonstrated moderate reliability (< 8% CV; good-moderate ICC) across both conditions. Perceptual ratings had poorer reliability during the Control condition (12–45% CV; poor ICC) than the Patrol condition (7–16% CV; good ICC).

**Conclusions:**

The test–retest reliability of metabolic and cardiovascular variables was high and relatively consistent during load carriage. Respiratory responses demonstrated moderate test–retest reliability; however, as the SWC differed with load carriage tasks, such data should be interpreted independently across loads. Perceptual measures demonstrated poor to moderate reliability during load carriage, and it is recommended that they only be employed as secondary measures.

**Graphical abstract:**

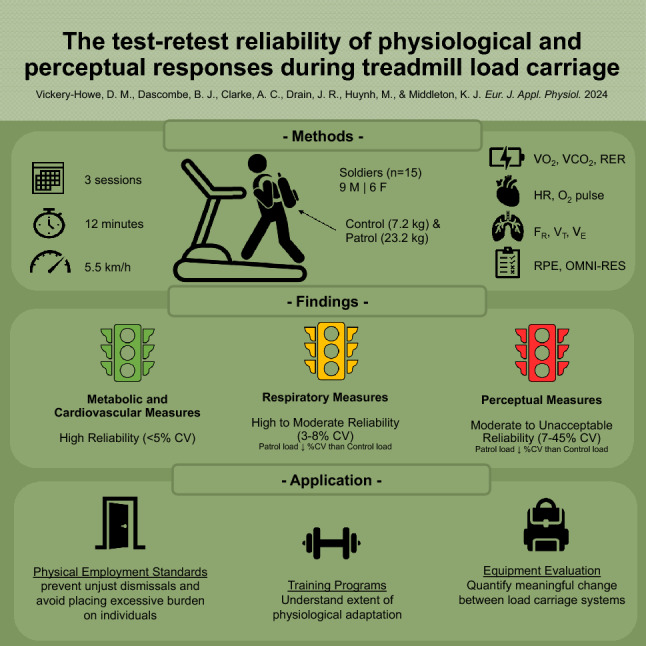

## Introduction

Load carriage is essential in physically demanding occupations such as the military and emergency services (Knapik et al. 2004; Taylor et al. 2016). Consequently, load carriage is an increasingly investigated research area due to both the importance and diverse nature of task requirements (Faghy et al. 2022). Within the military, the type, amount, and distribution of equipment carried by personnel vary due to several factors including role, mission requirements, and environment. However, general load echelons have been provided to help define the equipment and associated external loads necessary for mission types, typically being: Patrol load (~ 23 kg), fighting load (~ 30 kg), approach march load (~ 38 kg), and emergency approach march load (≥ 44 kg) (Department of the Army (US) 2022; Drain et al. 2017; North Atlantic Treaty Organisation 2009). Understanding the physiological strain from load carriage is important because it may impact marching performance or a soldier’s performance within a subsequent task. Additionally, measuring physiological responses to load carriage can support the evaluation of equipment and configurations (Polcyn et al. 2000) as well as occupational assessments (Reilly et al. 2019) and training interventions (Wills et al. 2020).

Assessing the test–retest reliability of physiological responses captures both the within-subject biological variation and technical variation of instrumentation (Bagger et al. 2003). Test–retest reliability can be influenced by various constraints, including individual (e.g., fitness, training, fatigue, mood), task (e.g. load distribution, rest time interval), and environmental (e.g. terrain, temperature, noise) (Newell 1986). Currently, research that has explored repeated trials using the portable gas analysis system of the MetaMax 3B is limited (Macfarlane and Wong 2012; Vogler et al. 2010). During laboratory testing, when assessed against a metabolic simulation system, the MetaMax 3B demonstrated very strong correlations (ICC = 0.996–1.00) across repeated trials for various gas-exchange variables that were within a representative physiological range (Macfarlane and Wong 2012; Vogler et al. 2010). Further, a single human study in elite youth rowers that undertook a progressive incremental exercise test on a rowing ergometer reported trivial between-session differences and a high level of reliability (2.3–4.5% coefficient of variation [CV]) when using the MetaMax 3B to quantify oxygen consumption ($\dot{V}{\text{O}}_{2}$), carbon dioxide production ($\dot{V}{\text{CO}}_{2}$), ventilation ($\dot{V}_{{\text{E}}}$), and respiratory exchange ratio (RER) (Vogler et al. 2010). However, load carriage introduces a unique challenge to the cardiorespiratory system, and it is unclear whether torso-borne load impacts the test–retest reliability of physiological responses as measured by the MetaMax 3B.

Separately, respiratory function has been shown to be affected by load carriage (Armstrong et al. 2019; Dominelli et al. 2012; Faghy et al. 2022; Phillips et al. 2016), particularly where the load is typically distributed across the anterior and posterior sides of the torso as well as the hands in military settings. Torso-borne load can compress the chest and lungs, resulting in additional resistance that must be overcome during minute ventilation ($\dot{V}_{{\text{E}}}$) (Armstrong et al. 2019; Faghy et al. 2022; Phillips et al. 2016). Armstrong et al. (2019) demonstrated that wearing a body armour system (10.9 kg) reduced pulmonary function (forced vital capacity and forced expiratory volume in one second) during rest, as well as resulting in higher $\dot{V}_{{\text{E}}}$, respiratory frequency (*F*_R_), and tidal volume (*V*_T_) during loaded marching (> 45 kg). Similar respiratory responses have been demonstrated during load carriage with pack-borne loads (Dominelli et al. 2012). Therefore, the introduction of external load results in changes in respiratory responses during such tasks. Factors to consider during repeated load carriage bouts, as compared with unloaded exercise, encompass fatigue stemming from the load-carrying task (maintaining consistent sequencing), training effects, and familiarity with load carriage (ensuring participants are habituated to load carriage). It is also essential to maintain consistency in the clothing and footwear worn across sessions, avoid periods where physical and mental fatigue might be present, as well as to ensure the proper fit and consistent adjustment of the external load. Therefore, while the impact of vest and pack loads on respiratory responses has been established, it remains unclear whether these effects are reliable across multiple sessions.

Due to the broad spectrum of characteristics to evaluate, load carriage research has been commonly conducted in a controlled laboratory environment on a single occasion (Drain et al. 2012; Knapik et al. 2004; Macfarlane 2001). The interpretation of these results impacts on subsequent energy expenditure estimates used to inform practical nutrition and recovery requirements, as well as setting physical employment standards and assessing new equipment and technologies. To accurately assess the physiological and perceptual demands of load carriage activities, it is essential to understand the typical variation between sessions in order to quantify what constitutes a meaningful difference (i.e. smallest worthwhile change [SWC]) between trials and conditions. Therefore, the aim of this study was to determine the between-session test–retest reliability of physiological and perceptual responses during treadmill-based load carriage.

## Materials and methods

### Participant information

Fifteen participants, including six female (mean ± SD; age: 23.0 ± 3.1 years; height: 1.68 ± 0.04 m; body mass: 66.4 ± 6.8 kg; load carriage experience: 9.2 ± 4.8 months) and nine male (27.2 ± 6.4 years; 1.79 ± 7.8 m; 84.2 ± 14.2 kg; load carriage experience: 24.0 ± 25.3 months) soldiers, were recruited from the Australian Defence Force School of Signals. All participants had completed basic military training and reported no known neuromuscular injuries or respiratory tract infections in the previous six months. All procedures were approved by the Department of Defence and Veterans’ Affairs Human Research Ethics Committee (Ethics #302-20) and reciprocal approval granted by the La Trobe University Human Ethics Committee (Ethics #302-20 DDVA HREC). Written informed consent was obtained from the participants prior to commencement.

### Protocol overview

Familiarisation of at least six minutes was conducted on an AMTI dual-belt (front and back) instrumented treadmill (Watertown, MA, USA), including Control (7.2 kg) and loaded (23.2 kg and 35.2 kg) walking between 4 and 6 km h^−1^ (Meyer et al. 2019). Three experimental sessions were completed, each separated by one week. Within each session, two twelve-minute walking trials were completed on the treadmill at 5.5 km h^−1^, with participants carrying either 7.2 kg (Control) or 23.3 kg (Patrol) loads as per the Australian Army baseline physical employment standard forced march assessment. Trials were separated by twelve minutes of passive rest. The Control condition was routinely performed before the Patrol condition to eliminate any influence of the higher exercise intensity evoked by load carriage. The Control condition included participants wearing a standard physical training uniform (shorts, t-shirt) with approved Australian Army combat boots (2 kg), and a portable metabolic system (2 kg) on the torso while also holding a replica F88 Austeyr (3.2 kg) in both hands. The Patrol condition consisted of the Control condition, with the addition of a weighted vest that distributed the additional weight evenly between left and right (using 1 kg blocks), and front and back (10 kg at front; 6 kg at back), which is representative of an in-field Patrol order distribution.

Expired respiratory gases were collected through a Hans Rudolf face-mask that was connected to a MetaMax 3B portable metabolic system (Cortex Inc., Germany). The MetaMax 3B system was turned on 60 min prior to calibration for volume and flow, and gas concentration measures. The calibration included (i) the input of barometric pressure, (ii) calibrating the gas analyser using a reference gas (15% O_2_, 5% CO_2_, BAL. N_2_; tolerance 1%, Cortex Inc., Germany) and sampling ambient air, and (iii) a flow calibration conducted using a standardised 3-L syringe (Hans Rudolph Inc., USA) at 2 to 4 and − 2 to − 4 L s^−1^. Data collection and therefore measurement of devices began within 15 min of the calibration being conducted. Heart rate was recorded using a chest strap (T31 coded, Polar Electro, Finland) that was collected through the MetaMax 3B. Prior to each trial, the MetaMax 3B was fit to the participant and allowed time to acclimate until $\dot{V}{\text{O}}_{2}$ was below 0.5 L min^−1^. During each trial, the MetaMax 3B continuously measured respiratory variables (*F*_R_, *V*_T_, $\dot{V}_{{\text{E}}}$), metabolic demands ($\dot{V}{\text{O}}_{2}$ [absolute and relative to body mass], $\dot{V}{\text{CO}}_{2}$, RER), and cardiovascular function (heart rate [HR], oxygen pulse [O_2_ pulse]) for the 12 min of walking. All expired gases were sampled using breath-by-breath measures. The final three minutes of data were averaged for inclusion within the analyses to ensure that the responses were reflective of a physiological steady state. Perceptual measures included Rating of Perceived Exertion (RPE; 6–20 scale) (Borg 1998) and the Omnibus Resistance Exercise Scale (OMNI-RES; 0–10 scale) (Robertson et al. 2003) which were explained to participants prior to experimental testing. The RPE scale was characterised as follows: “a score of 6 indicates no exertion at all as if you are resting, while a maximum score of 20 signifies maximal exhaustion and the most challenging exercise you have ever done”. Immediately following each 12-min trial, the participant was asked “how hard do you feel like you were working?” The OMNI-RES scale was used to measure the general impact of the total load carried and was characterised as follows: “a score of 0 indicates no load that is extremely easy, while a maximum score of 10 is extremely hard and the heaviest thing you have carried”, with participants asked, “how difficult was it to carry that load?” immediately following each 12-min trial.

To ensure consistency throughout the three laboratory visits, each testing session was scheduled on the same time and day of the week to align with weekly job schedule demands; undertaken in a consistent environmentally controlled laboratory environment with minimal noise (e.g. no music, no talking during testing trials); structured using standardised rest time interval between experimental trials; and repeated using the same sequence, load distribution and physical fit of load across the three sessions. Additionally, the same MetaMax 3B unit and researcher was used across the three sessions to account for inter-device and inter-researcher variation (Hopkins 2000).

### Statistical analysis

Descriptive statistics (mean ± standard deviation) were calculated for each physiological variable across the two load conditions (Control and Patrol) for each of the three sessions. Reliability was determined by coefficient of variation (CV), intra-class correlation coefficients (ICC_[2,1]_), smallest worthwhile change (SWC), and standard error of the measurement (SEM). CV thresholds have been defined for acceptable reliability, with results interpreted as either highly reliable (≤ 5.0%) or moderately reliable (5.1–10.0%), with CV > 10.0% being classified as unacceptable reliability. ICC results were interpreted as per Koo and Li (2016) using the following qualitative descriptors: poor (< 0.50), moderate (0.50–0.74), good (0.75–0.90), and excellent (> 0.90). SWC was calculated by 0.2 × between subject standard deviation for each condition (Hopkins and Batterham 2016). Outliers with a *z* score greater than ± 2.58 were removed (*n* = 9 data points were removed). The residuals of all physiological measures did approximate a normal distribution (as assessed by *Q*–*Q* plots and Kolmogorov–Smirnov tests). All statistical analyses were conducted using the jamovi statistical package (Version 2.2.5, the jamovi project, 2022).

## Results

Reliability statistics are presented in Table 1. Reliability was better in the Patrol condition than the Control condition for respiratory variables (Control: 5.5–8.0%, Patrol: 3.4–5.2% CV), metabolic demands (Control: 2.9–4.7%, Patrol: 2.2–3.7%), and perceptual measures (Control: 12.4–45.5%, Patrol: 7.4–16.1%). In contrast, the reliability of cardiovascular measures was better in the Control (3.1–3.5%) than the Patrol condition (7.4–16.1%). Between-session intra-class correlations for the Control condition were *poor* for RPE and OMNI-RES ratings (ICC_[2, 1]_ = 0.47–0.48), *moderate* for F_R_, relative $\dot{V}{\text{O}}_{2}$, RER, and HR (ICC_[2, 1]_ = 0.52–0.70), *good* for *V*_T_, $\dot{V}_{{\text{E}}}$, absolute $\dot{V}{\text{O}}_{2}$, and $\dot{V}{\text{CO}}_{2}$ (ICC_[2, 1]_ = 0.72–0.88), and *excellent* for O_2_ pulse (ICC_[2, 1]_ = 0.93). Between-session intra-class correlations for the Patrol condition were classified as *moderate* for relative $\dot{V}{\text{O}}_{2}$, RER, and HR (ICC_[2, 1]_ = 0.60–0.74) and *good* for *F*_R_, *V*_T_, $\dot{V}_{{\text{E}}}$, absolute $\dot{V}{\text{O}}_{2}$, $\dot{V}{\text{CO}}_{2}$, O_2_ pulse, RPE, and OMNI-RES (ICC_[2, 1]_ = 0.79–0.88).

**Table 1:**
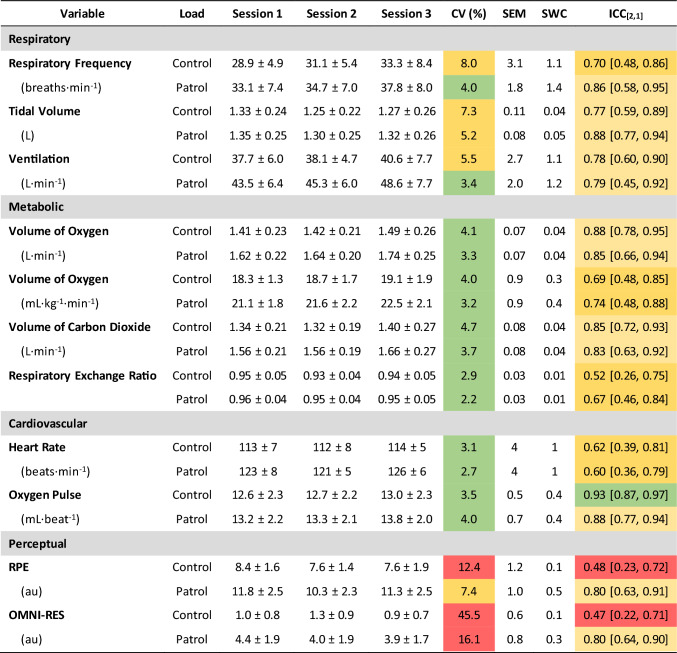
Mean ± standard deviation and reliability statistics for physiological measures across two conditions (Control and Patrol) and three sessions

CV has been colour coded by highly reliable (≤ 5.0%, green), moderate reliability (5.1–10.0, yellow), and unacceptable reliability (> 10.0, red); and ICC poor (< 0.50, red), moderate (0.50–0.74, dark yellow), good (0.75–0.90, light yellow), and excellent (> 0.90, green)

*CV* coefficient of variation, *ICC* intra-class correlation coefficient, *OMNI-RES* omnibus resistance exercise scale, *RPE* rating of perceived exertion, *SEM* standard error of the measurement, *SWC* smallest worthwhile change

## Discussion

The aim of this study was to determine the test–retest reliability of physiological and perceptual responses during treadmill-based load carriage at Control and Patrol loads. Overall, respiratory variables demonstrated moderate-good ICC (3–8% CV), while the metabolic and cardiovascular variables demonstrated moderate-excellent ICC (≤ 5% CV). Perceptual variables showed good-poor ICC (7–45% CV). As such, respiratory, metabolic, and cardiovascular variables appear to possess acceptable reliability at both different load conditions, and they can be confidently applied in practice from a single testing session to set physical employment standards, develop work-rest tables, or evaluate the impact of new equipment. Perceptual measures should be utilised with caution due to the varied reliability across measures, loads, and sessions, and be employed either to support the interpretation of physiological responses, or when no other physiological measures are able to be undertaken.

Firstly, the respiratory variables demonstrated high test–retest reliability (3–5% CV; good ICC) for the Patrol condition and moderate reliability for the Control condition (5–8% CV; good-moderate ICC; larger SEM). It appears the increasing intensity and the stimulus of torso-borne load elicits a more consistent respiratory response (Romer and McConnell 2004). While a previous study reporting on the reliability of the MetaMax 3B has reported lower CV (3.5% CV), measures for $\dot{V}_{{\text{E}}}$ during unloaded exercise (Vogler et al. 2010), it should be acknowledged that this was assessed on a rowing ergometer and not treadmill walking exercise. Hence, any interpretation of these reliability measures are limited by differences in both load and exercise modality. As soldiers are conditioned to complete military tasks such as load carriage, this experience and targeted training may increase task efficiency and reduce the variability in physiological response during loaded walking (Orr and Pope 2015; Wills et al. 2020). The greater external load may also perturb the system whereby work may be conducted within a smaller range in an attempt to limit centre of mass displacement when compared with a lighter load (Hoolihan et al. 2022; Liew et al. 2020). It appears the additional elastic and inertial forces due to the chest restriction of load carriage (Armstrong et al. 2019; Peoples et al. 2016) resulted in a more reliable response. Meaningful changes in respiratory responses due to load carriage should be assessed independently for lighter and heavier loads.

Metabolic and cardiovascular variables were highly reliable (< 5% CV; good-moderate ICC) for both the Control and Patrol conditions. Between-session differences reflect a combination of technical and biological variability (Armstrong and Costill 1985; Hopkins 2000). The between-session variation is not fully explained by the technical variation of the MetaMax 3B unit, as reported simulated metabolic outputs are smaller in magnitude and more reliable for $\dot{V}{\text{O}}_{2}$, $\dot{V}{\text{CO}}_{2}$, $\dot{V}_{{\text{E}}}$, and RER (Macfarlane and Wong 2012; Vogler et al. 2010). Therefore, biological variation likely explains the majority of the higher between-session variability reported in this study when compared to that calculated from metabolic gas simulators. Importantly, the reliability in the current study is similar to previous research (2–5% CV, excellent-good ICC) for metabolic responses taken during rowing (Vogler et al. 2010), and cardiovascular responses during rest, walking, and jogging (Engström et al. 2012; Montes and Navalta 2019; Nunan et al. 2009). In agreeance with previous studies, the constraint of load carriage during treadmill walking does not appear to impact the reliability of metabolic and cardiovascular responses through affecting the magnitude of technical and biological variation (Engström et al. 2012; Macfarlane and Wong 2012; Montes and Navalta 2019; Nunan et al. 2009; Vogler et al. 2010). Accordingly, metabolic and cardiovascular results from load carriage studies involving a single trial can be confidently applied in practice, with meaningful differences able to be identified utilising the CV and SWC data reported within this study.

Across the multiple sessions, the perceptual measures demonstrated a level of unacceptable test–retest reliability (12–45% CV; poor ICC) for the Control condition, though this was improved for the Patrol condition (7–16% CV; good ICC). Other studies have reported contrasting results whereby the reliability of perceptual measures decrease with increases in work intensity (Herman et al. 2006; Lamb et al. 1999). The Patrol condition may have similar effects of constraining the physical work with the torso-borne load when compared to the Control condition for a more consistent response. RPE (as measured on a 6–20 scale) demonstrated poor reliability (7.35–12.4% CV; 0.48–0.80 ICC), despite presenting with a reasonably small SWC (0.11–0.50). As this scale uses increments of one unit, further consideration may be required when interpreting a meaningful change. Separately, the OMNI-RES scale was developed and validated to provide a perceived intensity for resistance exercise (Robertson et al. 2003), and may not be appropriate for monitoring load carriage activities. Additionally, utilising CV as a measure of reliability for the OMNI-RES scale may be more sensitive to changes in the mean because the mean is close to zero. For perceptual measures, the restriction of arm swing with the weapon in the Control condition may have been perceived as increasing load carriage effort to different extents. This variability in responses could be attributed to the fact that the additional 16 kg load in the Patrol condition might have outweighed any discomfort associated with the weapon. The variability in perceptual data could also be due to various psychobiological factors that may influence an individual’s perception of exertion and load within a single session. As such, subjective perceptual measures demonstrate greater variability and poorer reliability than the physiological measures, and this needs to be considered when employing such measures to quantify the perceived demands of load carriage tasks.

Taken together, measures of reliability (i.e. CV and ICC) and meaningful changes (i.e. SWC and SEM) can serve as valuable metrics when interpreting physiological data from load carriage tasks that may be used to inform, among other things, physical employment standards, work-rest tables, and equipment evaluation. For example, in the assessment of a load carriage system, it is crucial for end-users to establish whether observed differences are meaningful or not. In the present study, the CV value of 4% for $\dot{V}{\text{O}}_{2}$ can be utilised to inform this when assessing across a wide range of loads. Vine and colleagues (2022) investigated the metabolic responses with a Douglas bag for load carriage tasks of between 30 and 76 kg, reporting a ~ 1% increase in metabolic cost for each 1 kg increment in added load mass. From our data, it can be interpreted that for load configurations of ≤ 4 kg difference, it is unlikely to observe clear differences in metabolic cost due to the variability in the measure. An additional application involves developing physical employment standards, ensuring personnel can meet the demands of their job tasks (Reilly et al. 2019). Ecologically valid measures of load carriage task demands are crucial for reflecting them in assessments, directly influencing a soldier’s employability. To maintain fairness, it is imperative to scientifically defend the measurement of load carriage demands, preventing unjust dismissals or avoiding placing excessive burden on individuals in job categories. Further, researchers and practitioners can employ SWC values to quantify: (a) the extent of adaptation to training programmes, helping understand how individuals are physiologically adapting to the load, and (b) meaningful changes between various load carriage systems that do not reflect measurement error.

A limitation of this study was the order effect, with the Control condition always being completed before the Patrol condition. Considering that the Patrol condition was more reliable, there is a potential familiarisation effect to the testing scenario. However, considering that only the final 3-min of each stage were included for analysis, it is expected that any familiarisation effects would be minimal as they have been reported to typically disappear after six minutes (Meyer et al. 2019). Further, slight differences in chest compression as a result of securing the vest and harness could have impacted respiratory mechanics and provided a source of variation between sessions. While this was consistently applied by the same researcher, interface pressure was not measured. Future studies should incorporate a pressure feedback unit to keep a consistent compressive load on the chest (Peoples et al. 2016). Future research is required to assess different walking speeds and loads outside of the range assessed in this study to evaluate whether these responses are consistent.

The test–retest reliability of physiological responses during treadmill walking with external loads was demonstrated to be highly reliable (< 5% CV) for all metabolic and cardiovascular measures, as well as both F_R_ and $\dot{V}_{{\text{E}}}$ for the Patrol load. Moderate-to-poor reliability was demonstrated for all other respiratory measures and all perceptual measures. While it is important to note that a degree of biological and technical variation is expected between trials, both the CV and SWC are appropriate measures that can be employed to determine meaningful changes in physiological response during load carriage tasks. Together, these reliability data can support the interpretation of physiological assessments related to training adaptations, equipment evaluations, tasks, populations, or other factors to ensure that any observed differences are interpreted appropriately.

## Data Availability

All data pertaining to this research article are included in this manuscript.

## Abbreviations

$\dot{V}{\text{CO}}_{2}$: Carbon dioxide production
CV: Coefficient of variation
DDVA: Department of Defence and Veterans’ Affairs
HR: Heart rate
HREC: Human Research Ethics Committee
ICC: Intra-class correlation coefficients
kg: Kilogramme
km·h^−^^1^: Kilometres per hour
L: Litres
N: Number
OMNI-RES: Omnibus resistance exercise scale
$$\dot{V}{\text{O}}_{2}$$: Oxygen consumption
O_2_ pulse: Oxygen pulse
RPE: Rating of perceived exertion
RER: Respiratory exchange ratio
F_R_: Respiratory frequency
s: Second
SD: Standard deviation
SEM: Standard error of the measurement
SWC: Smallest worthwhile change
V_T_: Tidal volume
$$\dot{V}_{{\text{E}}}$$: Ventilation

## References

[CR1] Armstrong LE, Costill DL (1985). Variability of respiration and metabolism: responses to submaximal cycling and running. Res Q Exerc Sport.

[CR2] Armstrong NCD, Ward A, Lomax M, Tipton MJ, House JR (2019). Wearing body armour and backpack loads increase the likelihood of expiratory flow limitation and respiratory muscle fatigue during marching. Ergonomics.

[CR3] Bagger M, Petersen PH, Pedersen PK (2003). Biological variation in variables associated with exercise training. Int J Sports Med.

[CR4] Borg G (1998). Perceived exertion and pain scales.

[CR5] Department of the Army (US) (2022) Foot Marches (ATP 3-21.18)

[CR6] Dominelli PB, William Sheel A, Foster GE (2012). Effect of carrying a weighted backpack on lung mechanics during treadmill walking in healthy men. Eur J Appl Physiol.

[CR7] Drain JR, Aisbett B, Lewis M, Billing DC (2017). The Pandolf equation under-predicts the metabolic rate of contemporary military load carriage. J Sci Med Sport.

[CR8] Drain J, Orr R, Attwells RL, Billing DC (2012) Load carriage capacity of the dismounted combatant—a commander’s guide

[CR9] Engström E, Ottosson E, Wohlfart B, Grundström N, Wisén A (2012). Comparison of heart rate measured by polar RS400 and ECG, validity and repeatability. Adv Physiother.

[CR10] Faghy MA, Shei RJ, Armstrong NCD, White M, Lomax M (2022). Physiological impact of load carriage exercise: current understanding and future research directions. Physiol Rep.

[CR11] Herman L, Foster C, Maher MA, Mikat RP, Porcari JP (2006). Validity and reliability of the session RPE method for monitoring exercise training intensity. S Afr J Sports Med.

[CR12] Hoolihan B, Wheat JS, Vickery-Howe DM, Dascombe BJ, Middleton KJ (2022). The effect of external loads and biological sex on coordination variability during load carriage. Gait Posture.

[CR13] Hopkins WG (2000). Measures of reliability in sports medicine and science. Sports Med.

[CR14] Hopkins WG, Batterham AM (2016). Error rates, decisive outcomes and publication bias with several inferential methods. Sports Med.

[CR15] Knapik JJ, Reynolds KL, Harman E (2004). Soldier load carriage: historical, physiological, biomechanical, and medical aspects. Mil Med.

[CR16] Lamb KL, Eston RG, Corns D (1999). Reliability of ratings of perceived exertion during progressive treadmill exercise. Br J Sports Med.

[CR17] Liew BXW, Morris S, Netto K (2020). Trunk–pelvis coordination during load carriage running. J Biomech.

[CR18] Macfarlane DJ (2001). Automated metabolic gas analysis systems—a review. Sports Med.

[CR19] Macfarlane DJ, Wong P (2012). Validity, reliability and stability of the portable cortex Metamax 3B gas analysis system. Eur J Appl Physiol.

[CR20] Meyer C, Killeen T, Easthope CS, Curt A, Bolliger M, Linnebank M, Zörner B, Filli L (2019). Familiarization with treadmill walking: how much is enough?. Sci Rep.

[CR21] Montes J, Navalta JW (2019). Reliability of the polar T31 uncoded heart rate monitor in free motion and treadmill activities. Int J Exerc Sci.

[CR22] Newell KM (1986). Constraints on the development of coordination. Motor development in children: aspects of coordination and control.

[CR23] North Atlantic Treaty Organisation (2009) Optimizing operational physical fitness, vol TR-HFM-080

[CR24] Nunan D, Gay D, Jakovljevic DG, Hodges LD, Sandercock GRH, Brodie DA (2009). Validity and reliability of short-term heart-rate variability from the polar S810. Med Sci Sports Exerc.

[CR25] Orr RM, Pope R (2015). Optimizing the physical training of military trainees. Strength Cond J.

[CR26] Peoples GE, Lee DS, Notley SR, Taylor NAS (2016). The effects of thoracic load carriage on maximal ambulatory work tolerance and acceptable work durations. Eur J Appl Physiol.

[CR27] Phillips DB, Stickland MK, Petersen SR (2016). Ventilatory responses to prolonged exercise with heavy load carriage. Eur J Appl Physiol.

[CR28] Polcyn AF, Bensel CK, Harman EA, Obusek JP (2000) The effects of load weight: a summary analysis of maximal performance, physiological and biomechanical results from four studies of load carriage systems. In: Soldier mobility: innovations in load carriage system design and evaluation

[CR29] Reilly T, Drain J, Blacker S, Sharp M, Hauret K (2019) HFM: combat integration: implications for physical employment standards

[CR30] Robertson RJ, Goss FL, Rutkowski J, Lenz B, Dixon C, Timmer J, Frazee K, Dube J, Andreacci J (2003). Concurrent validation of the OMNI perceived exertion scale for resistance exercise. Med Sci Sports Exerc.

[CR31] Romer LM, McConnell AK (2004). Inter-test reliability for non-invasive measures of respiratory muscle function in healthy humans. Eur J Appl Physiol.

[CR32] Taylor NAS, Peoples GE, Petersen SR (2016). Load carriage, human performance, and employment standards. Appl Physiol Nutr Metab.

[CR33] Vine CAJ, Coakley SL, Blacker SD, Doherty J, Hale BJ, Walker EF, Rue CA, Lee BJ, Flood TR, Knapik JJ, Jackson S, Greeves JP, Myers SD (2022). Accuracy of metabolic cost predictive equations during military load carriage. J Strength Cond Res.

[CR34] Vogler AJ, Rice AJ, Gore CJ (2010). Validity and reliability of the cortex MetaMax3B portable metabolic system. J Sports Sci.

[CR35] Wills JA, Drain J, Fuller JT, Doyle TLA (2020). Physiological responses of female load carriage improves after 10 weeks of training. Med Sci Sports Exerc.

